# Floral ontogeny of *Tachigali* (Caesalpinioideae, Fabaceae) species

**DOI:** 10.7717/peerj.13975

**Published:** 2022-09-08

**Authors:** Jamile Marques Casanova, Domingos Cardoso, Claudia Franca Barros, Haroldo Cavalcante de Lima, Karen L.G. De Toni

**Affiliations:** 1 Escola Nacional de Botânica Tropical, Instituto de Pesquisas Jardim Botânico do Rio de Janeiro, Rio de Janeiro, Rio de Janeiro, Brazil; 2 Diretoria de Pesquisas, Instituto de Pesquisas Jardim Botânico do Rio de Janeiro, Rio de Janeiro, Rio de Janeiro, Brazil; 3 Instituto de Biologia, Universidade Federal da Bahia, Salvador, Bahia, Brazil

**Keywords:** Floral development, Floral symmetry, *Sclerolobium*

## Abstract

**Background:**

The present ontogenetic study reveals variations throughout floral development in three morphologically representative species from the genus *Tachigali*, allowing a better understanding of floral organs diversity, flower symmetry and their homologies, especially in Fabaceae, a diverse family that exhibits a wide variation in floral architecture. *Tachigali* (Caesalpinioideae) corresponds to an important Neotropical legumes tree genus with 58 species in Brazil. Species of the genus *Sclerolobium* Vogel were incorporated in its circumscription, increasing the diversity of its floral morphology.

**Methods:**

This work aims to perform an ontogenetic study of *T. denudata*, *T. paratyensis* and *T. spathulipetala*, morphologically representative species of *Tachigali*, in order to describe the floral development and to better comprehend the floral morphology varieties among the species, using scanning electron microscopy.

**Results:**

We found the studied species to have floral buds with acropetal and helical development along the inflorescence axis; sepals and petals with helical development, varying the position of the primordia in the bud, according to the different species; stamens with unilateral development and carpel with adaxial curvature. These data correspond to original records of *Tachigali* ontogeny and contribute to an improved understanding of floral morphology and symmetry with data related to the zygomorphic and early development of the sepals and petals.

## Introduction

The Fabaceae family is one of the largest angiosperm families and figures among the most relevant groups for its economic and ecological importance (LPWG, 2017). This family is well known for its floral morphology, in particular its wide variety of shapes, as observed not only in the characteristics papilionaceous flowers, but also in actinomorphic, or asymmetric flowers observed among the group (Tucker, 2003). Studies of floral development have been helping the understanding of such morphological varieties and their homologies among Fabaceae flowers (Bruneau et al., 2014; Leite, Mansano & Teixeira, 2014; Leite et al., 2015; Prenner et al., 2015; Prenner & Cardoso, 2017). However, in Caesalpinioideae, a subfamily with a great variety in terms of floral architecture, many of its groups are poorly documented regarding floral ontogeny and development, mainly after its new circumscription (Tucker, Stein & Derstine, 1985; Tucker, 1988; Tucker, 1991; Tucker, 1992a; Tucker, 1996; Tucker & Kantz, 1997; Prenner, 2004; Marazzi & Endress, 2008), as seen for *Tachigali* Aubl. (Caesalpinioideae) (Casanova et al., 2020).

*Tachigali* is an important genus among the Neotropical arboreal leguminous flora (Silva & Lima, 2007), mainly owing to its ecological relevance as one of the most abundant genera of the Amazon rainforest (ter Steege et al., 2013). Within *Arapatiella* Rizzini & A. Mattos and *Jacqueshuberia* Ducke they form the Tachigali clade (Haston, Lewis & Hawkins, 2005). Most recently, the genus *Sclerolobium* Vogel has been circumscribed to *Tachigali* based on studies of wood morphology (Barreta-Kuipers, 1981; Macedo et al., 2014), pollen grain morphology (Graham & Barker, 1981) and external morphology of species (Silva & Lima, 2007; van Der Werff, 2008). According to Silva (2007), *Tachigali* genus covers tree species with simple or compound stipules paripinnate leaves. The inflorescences are in panicles with deciduous bracts. Its flowers vary from actinomorphic to zygomorphic, sessile to pedicellate, with cupular or tubular receptacle; all with five sepals, five petals and eight to 10 stamens. The gynoecium is stipitate, with the stipe attached to the center of the hypanthium or displaced laterally. Its fruits are of the cryptosamara type.

As a consequence of its circumscription, the variety of floral morphology within *Tachigali* has been expanded, currently including species with both actinomorphic and zygomorphic flowers with spatulated and/or filiform petals, tubular or cupular-shaped hypanthium, and ovary stipe placed in a central position, or laterally dislocated, in the hypanthium (van Der Werff, 2008), all of which has created some difficulty in understanding the relationships among the species within the group. Despite this wide variation, Casanova et al. (2020) in a recent work demonstrated that some aspects, such as unequal development of the hypanthium, are crucial for its floral symmetry which shows that their flowers are zygomorphic before anthesis and alternate between actinomorphic and zygomorphic after anthesis, owing to unique petals and stamens.

Comparative studies of external morphology and anatomy have helped us to understand the genus circumscription (Casanova et al., 2020); nonetheless, ontogenetic studies can shed new light by the addition of novel characters and, thereby, strengthen knowledge about the flower morphological diversity within *Tachigali* and among Caesalpinioideae (Tucker, 1992b; Tucker, 2003; Bruneau et al., 2014; Leite, Mansano & Teixeira, 2014; Leite et al., 2015; Prenner et al., 2015; Prenner & Cardoso, 2017). Therefore, the present work aims to perform an ontogenetic study of *T. denudata*, *T. paratyensis* and *T. spathulipetala*, three morphologically representative species from the *Tachigali*, which includes small and large flowers, variable in morphological terms. *Tachigali paratyensis* presents zygomorphic flowers, long pedicellate, with tubular hypanthium, spatulate petals, heteromorphic stamens and ovary stipe attached laterally to the hypanthium wall. *Tachigali spathulipetala* has actinomorphic flowers, also pedicellate, with cupular hypanthium, heteromorphic petals (spatulate and filiform), isomorphic stamens, and ovary stipe attached laterally to the hypanthium wall. *Tachigali denudata* has actinomorphic flowers, sessile to short pedicellate, with cupular hypanthium, filiform petals, isomorphic stamens, and ovary stipe in a central position in the hypanthium (Silva 2007 and Silva et al., 2016). These ontogenetical studies will promote a better understanding of the genus floral organs varieties, their symmetry, and homologies among Caesalpinoideae.

## Materials and Methods

Three species of the genus *Tachigali*, *Tachigali denudata* (Vogel) Oliveira-Filho, *T. paratyensis* (Vell.) H. C. Lima, and *T. spathulipetala* L. G. Silva, L. J. T. Cardoso, D. B. O. S. Cardoso & H. C. Lima, were chosen to study the floral ontogeny as they represent the floral morphology variation observed in the genus: small and large flowers with actinomorphic/zygomorphic symmetry, spathulate and/or linear petals, the ovary stipe arising in a central portion or laterally displaced in the hypanthium. Flower buds of *T. paratyensis* (RB 43559) were collected at the Arboretum of the Rio de Janeiro Botanical Garden and fixed in glutaraldehyde 2.5% in 0.1 M sodium phosphate buffer at pH 7.2 (Gabriel, 1982). *Tachigali spathulipetala* samples (RB 460994; RB spirit 1327; RB 459830; RB spirit 1329) were obtained from the Spirit Collection of the herbarium from the Rio de Janeiro Botanical Garden and fixed in ethanol 70%. *Tachigali denudata* (RB 659134) samples were taken from the personal collection of Professor Haroldo C. Lima, which were also preserved in ethanol 70%. Flower buds at different developmental stages were dissected with the Leica MZ16 stereomicroscope.

The material was then dehydrated in ethanolic series and, subsequently, in acetone (Bozzola & Russel, 1999, modified). Dehydration was concluded using the Bal-Tec Critical Point Dryer CPD 030. Samples were then fixed with double-sided carbon adhesive tapes to stubs and then covered with a thin layer of gold (~20 nm), using the Emitech K550X Sputter Coater. The material was analyzed with the Zeiss EVO 40 and JEOL JSM-6390LV scanning electron microscope.

To describe the type of aestivation, we followed the Endress (1994) classification.

## Results

**Figure 1:**
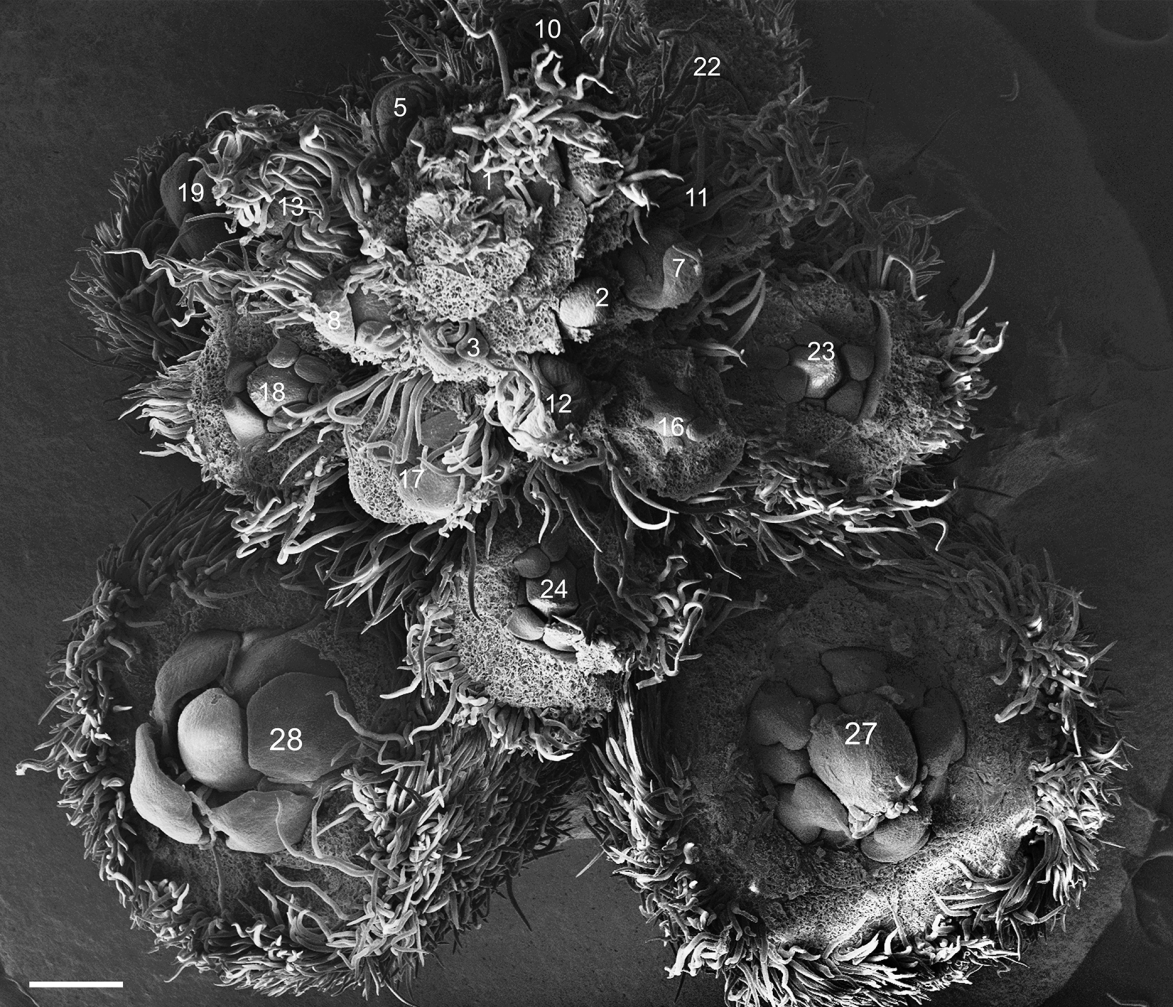
*Tachigali paratyensis* inflorescence. Apex showing the acropetal and helical growth of flower buds (1–28). Bar: 200 µm.

All analyzed species have paniculated inflorescence. Flowers and flower buds develop acropetally and helically along the inflorescence axis (Fig. 1). In the apical zone of the inflorescence, a bract protects each flower bud. The bracts develop before the floral primordium, covering the buds exclusively during the early stages, senescing as they mature and approach floral anthesis. The flower buds follow the basic pentameric plan of leguminous plants, containing five sepals, five petals, five stamens in a double whorl and one carpel. Because of differences detected in the developing patterns of sepals and petals (Table 1), results related to the ontogeny of the floral whorls were divided into two patterns.

**Table 1:** Summary of floral ontogeny results of the analysed species.

Species	Shared characters	Differential characters	Floral diagram
*Tachigali denudata*	Sepals: helical development, quincuncial aestivation; first one adaxial. Petals: helical development, slightly cochlear aestivation; first one abaxial and fifth adaxial. Androecium: unidirectional development. Gynoecium: adaxial carpel cleft, adaxial style bend.	Sepal: second abaxial; third lateral; fourth adaxial and fifth lateral; late trichomes. Petal: third abaxial and fourth lateral; filiform; early trichomes on the abaxial surface and on the carpel.	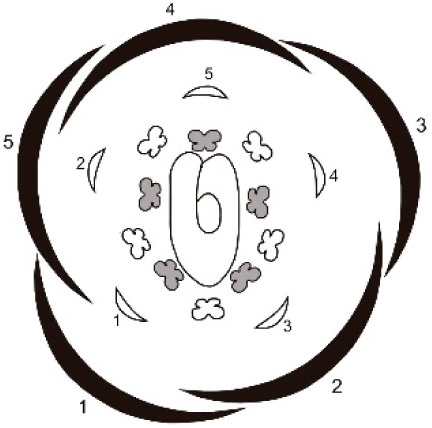
*Tachigali paratyensis*		Sepal: second adaxial, third and fourth in lateral position; fifth adaxial; early trichomes on the adaxial surface. Petal: third in lateral position and fourth abaxial; heteromorph (spatulate); late trichomes on the abaxial surface and on the carpel.	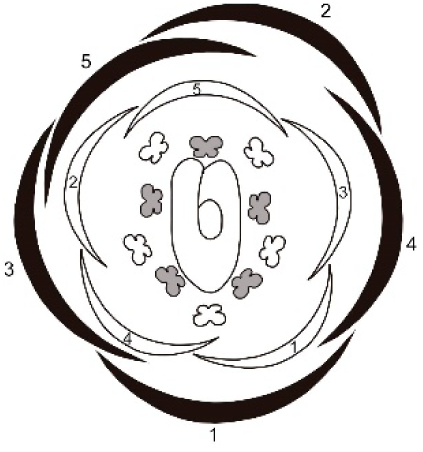
*Tachigali spathulipetala*		Sepal: second abaxial; third in lateral position and fourth adaxial; late trichomes in the sepals. Petal: third abaxial and fourth in lateral position; heteromorph (linear-lanceolate, spatulate); early trichomes on both surfaces and on the carpel.	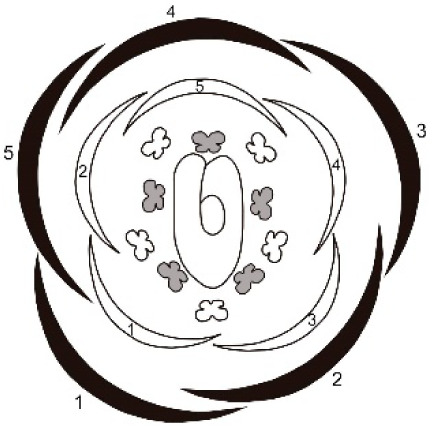

**Figure 2:**
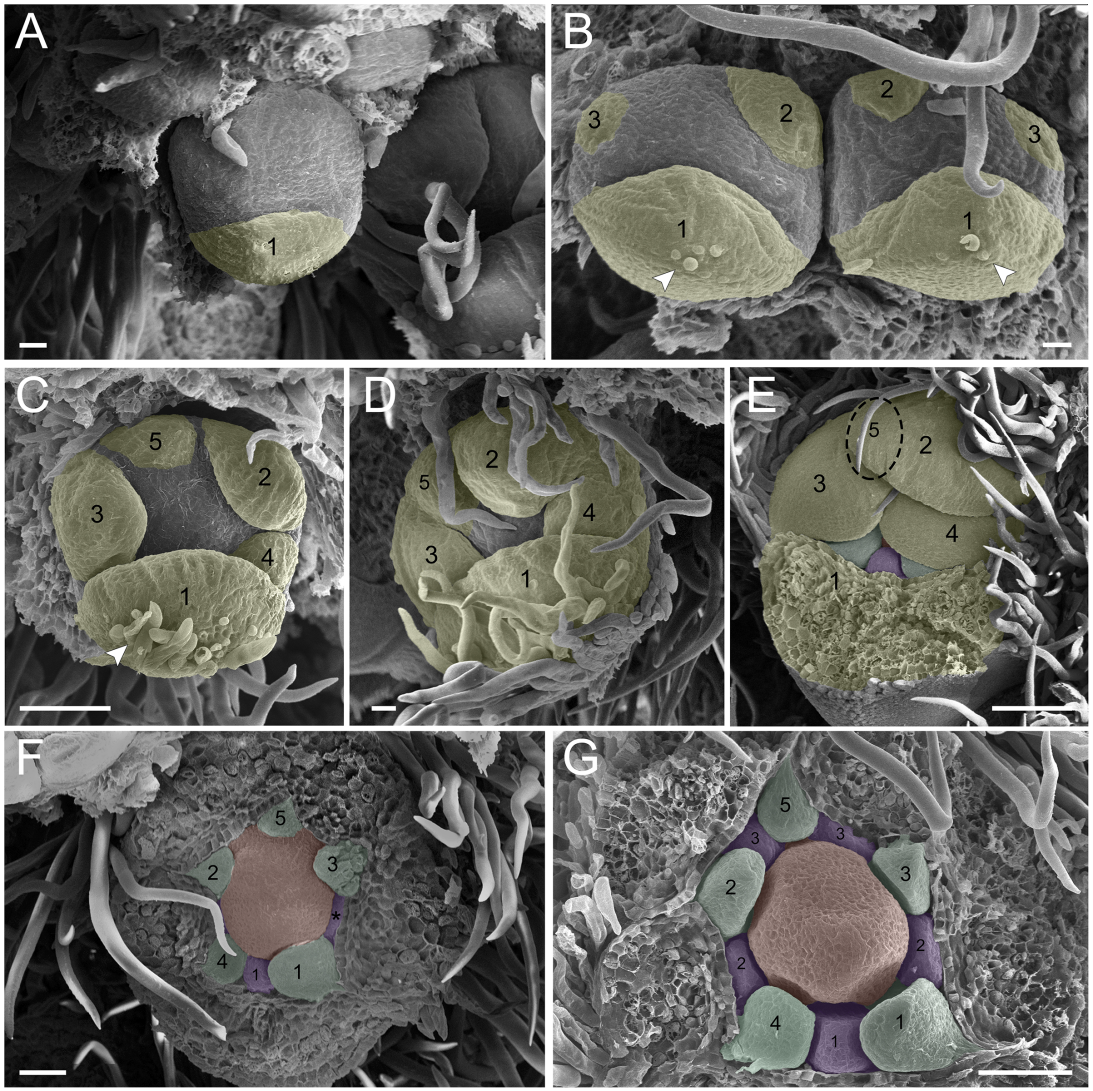
Floral ontogeny of *Tachigali paratyensis*. (A–G) Inflorescence axis at the top of the images. (A–D) Bracts removed; (E–G) bracts and sepals removed. (A) Floral primordium at the base of the bract; observe the formation of the first sepal (yellow) in the abaxial position. (B) Two floral primordia with three sepals initiated (yellow 1–3); emergence of simple trichomes indicated by arrowhead. (C) Three sepals in formation (yellow 1–3) and the primordia of the fourth (yellow 4) and fifth (yellow 5); simple trichomes indicated by arrowhead. (D) All sepals already set in the flower bud (yellow 1–5). (E) First sepal (yellow 1) removed, the other sepals (yellow 2–5) are arranged as quincuncial aestivation pattern. The dashline indicated the position of the five sepal under the sepals 2 and 3. (F) Five petals in formation (green 1–5), as well as the first stamen in abaxial position (purple 1) and the second stamen primordia (asterisk) carpel development initiation (orange). (G) Establishment of five petals (green 1–5) and antesepalous stamens (purple 1–3); carpel in development (orange). Bar: A, B, D and E = 20 µm; C and F = 50 µm.

**Figure 3:**
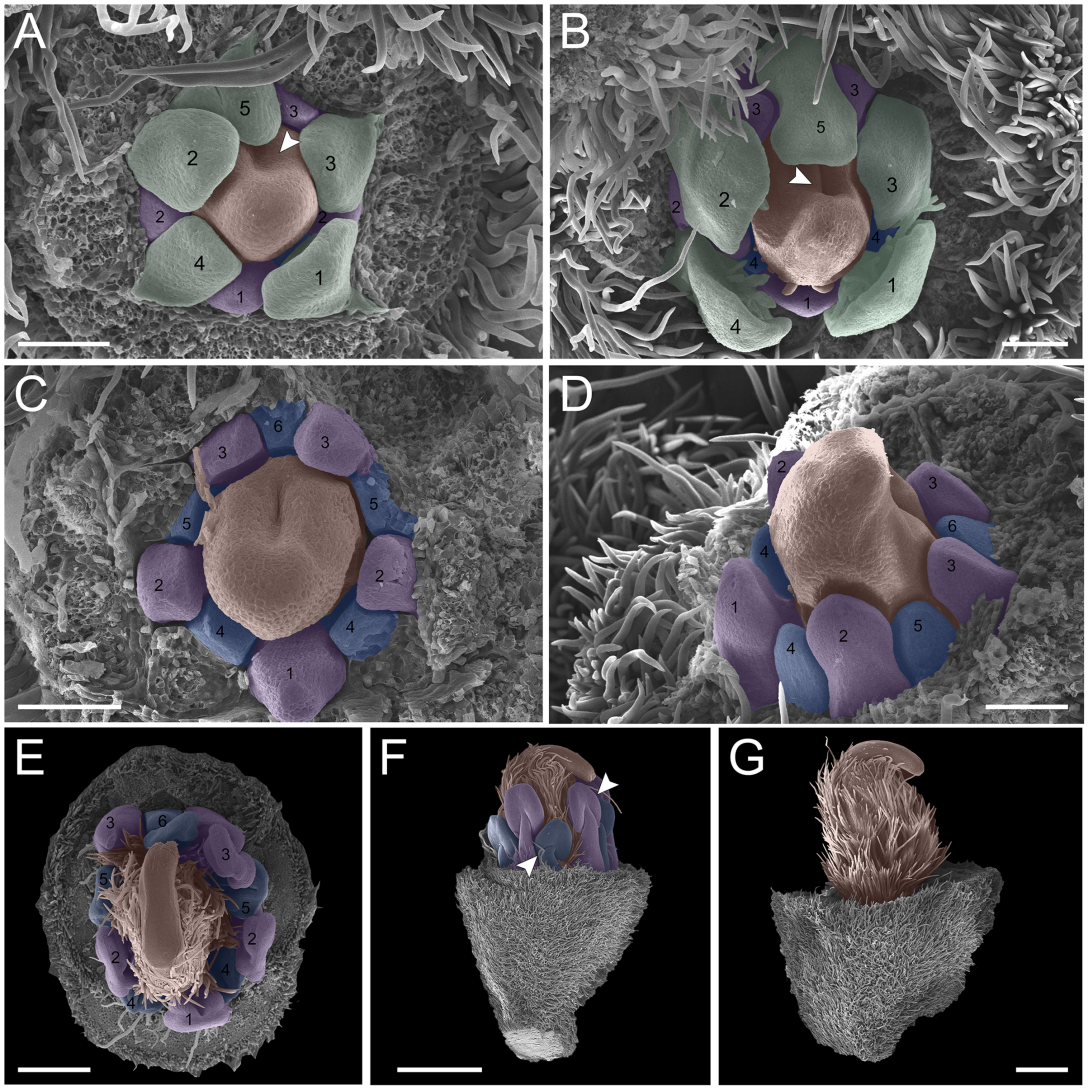
Floral ontogeny of *Tachigali paratyensis*. (A–C) Inflorescence axis at the top of the images. (A, B) Bracts and sepals removed. (C–F) Bracts, sepals and petals removed; (G) bracts, sepals, petals, and stamens removed. (A) Establishment of petals (green 1–5) and antesepalous whorl of stamens (purple 1–3), and the first antepetalous stamen primordium (blue); carpel in development (orange) with emergence of the carpel cleft (arrowhead). (B) Petals expanding (green 1–5); antesepalous whorl of stamens (purple 1-3) and antepetalous stamens primordia (blue 4); and carpel elongating (orange) with the closure of the carpel cleft (arrowhead). (C) Development of antesepalous stamens (purple 1–3) and antepetalous stamens primordia (blue 4–6); carpel elongating (orange). (D) Stamen development (purple 1–3; blue 4–6); carpel curvature (orange) towards the adaxial portion. (E) Established double whorl of stamens (purple 1–3; blue 4–6); style bent towards the adaxial portion (orange). (F) Side view of floral bud; arrowhead indicating the double whorl of stamens (purple and blue). (G) Side view of the flower bud showing only the gynoecium (orange). Bar: A–D = 100 µm; E = 500 µm; F, G = 200 µm.

In the first pattern, sepal development of *T. paratyensis* follows a helical pattern (Figs. 2A–2D). The first sepal is formed in the abaxial region close to the bract (Fig. 2A), followed by the second sepal in the adaxial region close to the inflorescence axis (Fig. 2B). The third sepal appears in a lateral position (Fig. 2B), as well as the fourth, which originates in the sequence (Fig. 2C); finally, the last sepal also appears in the adaxial region (Fig. 2D). In the next stage of development, it is evident that the first sepal, which was removed in Fig. 2E, has a completely outer position, as well as the second sepal (Figs. 2C–2E). They present a quincuncial aestivation type (Figs. 2D and 2E). At the beginning of sepal development, the formation of simple trichomes can be observed (Figs. 2B–2D). The petal development is helical (Figs. 2F and 2G; Figs. 3A and 3B). Similar to the sepal, the first petal is formed in the abaxial region (Fig. 2F), followed by the second sepal laterally placed and then by the third in the lateral opposite side (Fig. 2F). The fourth petal grows in the abaxial zone (Fig. 2F) and the fifth in the adaxial zone (Fig. 2F). The petals initiate their formation only after the setting of the five sepals (Figs. 2F and 2G) in an interspersed manner. The aestivation type is defined as cochlear (Figs. 3A and 3B). Moreover, it is important to highlight that both sepal and petal whorls might develop clockwise and counterclockwise (Fig. 2B).

In the androecium, the external whorl is formed by antesepalous stamens, which initiate their development soon after the beginning of petal formation (Figs. 2F and 2G). The antesepalous stamens follow a unidirectional pattern (Figs. 2F and 2G; Fig. 3A) with the first stamen in the abaxial region (Fig. 2F), followed by two stamens at a lateral position (Figs. 2F and 2G) and two in the adaxial region (Fig. 2G; Fig. 3A). After the setting of the outer whorl of the androecium, the formation of the inner whorl occurs (Fig. 3C). They present antepetalous stamens (Fig. 3C), which also follow a unidirectional pattern; however, two stamens appear in the abaxial region: two laterally placed and one in the adaxial region. Upon establishment of the carpel, the anthers begin to differentiate, first on the outer whorl of stamens and then on the inner whorl (Figs. 3D and 3E), establishing themselves at distinct levels (Fig. 3F). In the already differentiated anthers, it is possible to observe an extension of the dome-shaped connective.

The carpel initiates its development with the antesepalous stamens. The carpel cleft is adaxial, and it soon closes, curving towards the adaxial region (Figs. 3A–3D). In addition, the style prolongates towards the adaxial region, along with the differentiation of the anthers (Figs. 3E–3G).

**Figure 4:**
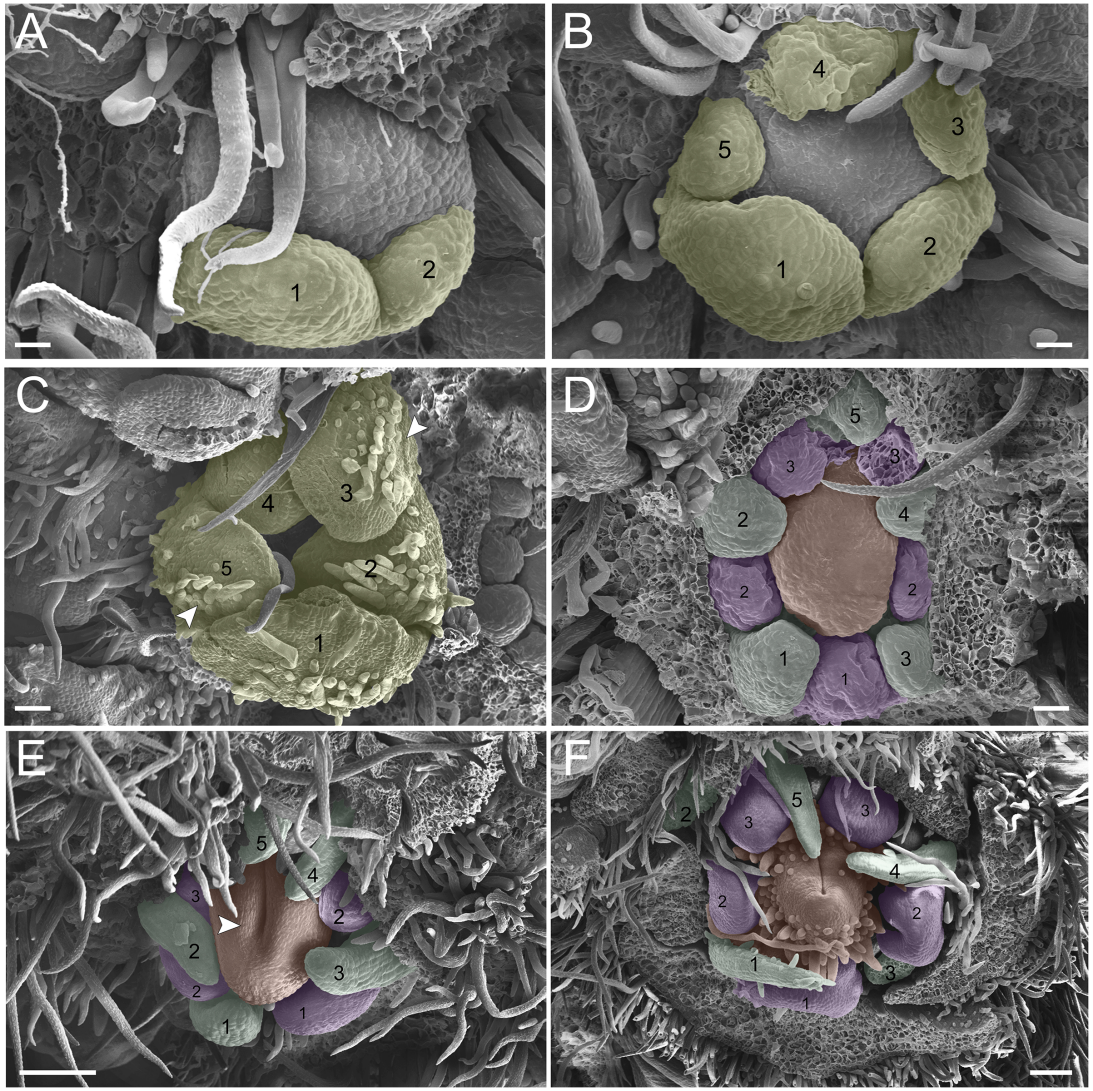
Floral ontogeny of *Tachigali denudata* (A–F). (A–F) Inflorescence axis at the top of images. (A–C) Bracts removed. (D–F) Bracts and sepals removed. (A) Floral primordium at the base of the bract; observe the primordia of the first and second sepal (yellow 1–2). (B) Emergence of the third, fourth and fifth sepals (yellow 3–5). (C) Five sepals (yellow 1–5); development of trichomes in the abaxial surface of the sepals (arrowhead). (D) Primordia of the five petals (green 1–5); primordia of the antesepalous stamens (purple 1–3); carpel development initiation (orange). (E) Filiform petals established (green 1–5); carpel in development (orange) with emergence of the carpel cleft (arrowhead). (F) Filiform petals established (green 1–5); carpel elongating (orange) with closure of the carpel cleft. Bar: A, B, D–F = 20 µm; C = 100 µm.

**Figure 5:**
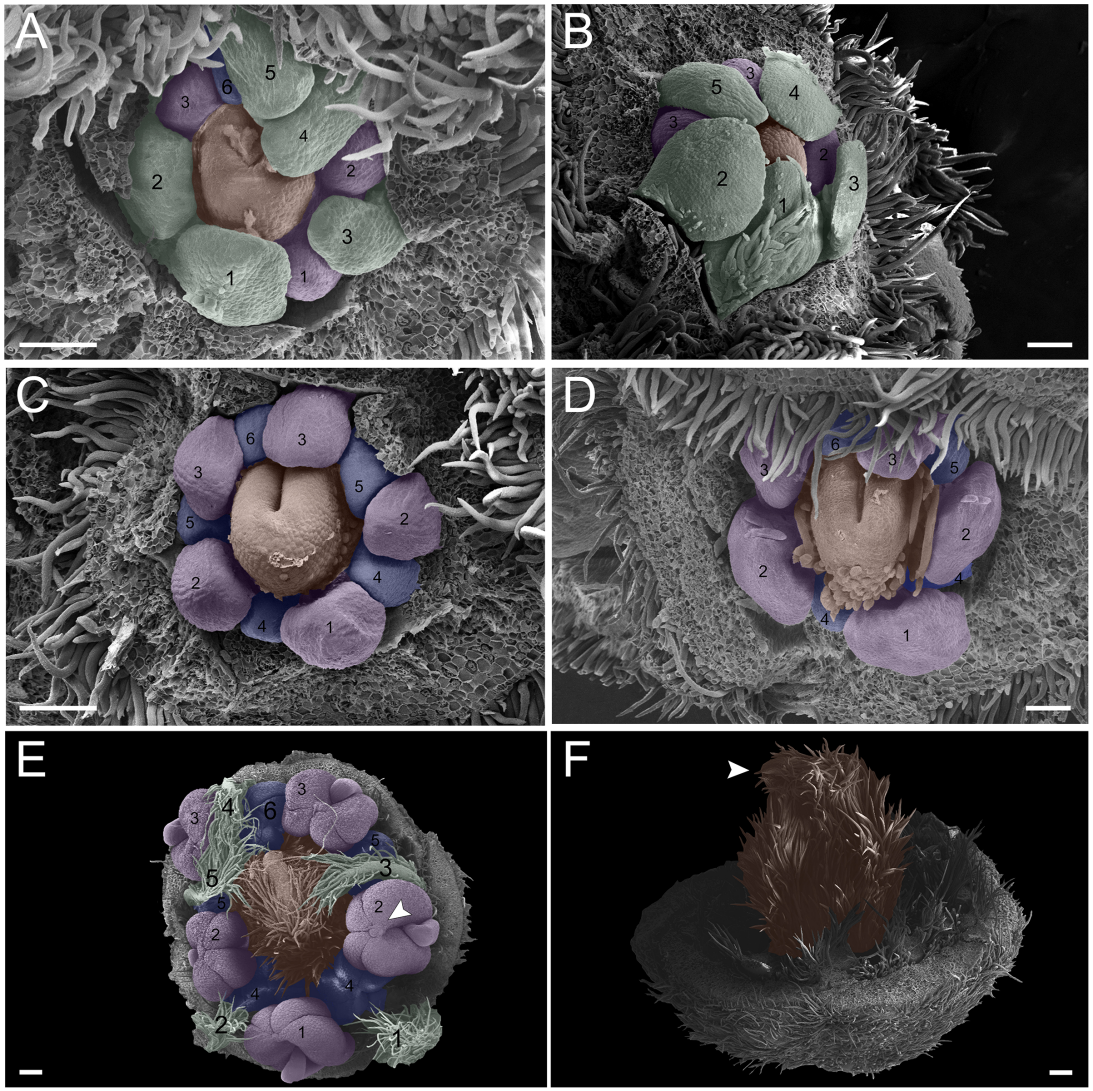
Floral ontogeny of *Tachigali spathulipetala* (A–D) and *Tachigali denudata* (E, F). (A–D) Inflorescence axis at the top of images. (A, B) Bracts and sepals removed. (C–E) Bracts, sepals and petals removed. (F) Bracts, sepals, petals, and stamens removed. (A, B) Filiform and spatulate petals (green 1–5); an antepetalous stamen primordium (blue 6); and carpel in development (orange). (C) Antepetalous stamens established (purple 1–3); antepetalous stamen primordia (blue 4–6); carpel in development (orange) with emergence of carpel cleft. (D) Anthers of the antesepalous stamens expanding (purple 1–3); and antepetalous stamen primordia (blue 4–6); and carpel (orange) elongating. (E) Anthers already formed on both androecium whorls (purple 1–3, blue 4–6); connective extension (arrowhead) style bent towards the adaxial portion. (F) Side view of the flower bud, only the gynoecium (orange); arrowhead pointing to stigma. Bar: A–D = 100 µm; E, F = 200 µm.

In the second pattern, for both *T. denudata* (Fig. 4; Figs. 5E and 5F) and *T. spathulipetala* (Figs. 5A–5D), the sepals are formed in a modified helical pattern. The first sepal emerges in the abaxial region (Fig. 4A), and the second emerges beside the former, also in the abaxial region (Figs. 4A and 4B). The third sepal develops laterally, the fourth in the adaxial side of the flower bud, and, finally, the fifth is formed in the lateral opposite side (Fig. 4B). They present a quincuncial aestivation type (Fig. 4C), and the trichomes only begin their development after settling of the sepals is concluded (Fig. 4C).

Petal formation also presents a helical pattern interspersed with the sepals (Fig. 4D). The first petal develops in the abaxial region of the flower bud, followed by the second laterally placed, the third in the abaxial region, the fourth also laterally, opposite to the second, and, finally, the fifth petal in the abaxial region (Fig. 4D). All *T. denudata* petals present a filiform shape (Figs. 4E and 4F), and don’t keep contact with each other, *i.e.*, the imbricate aestivation is not present. In contrast, *T. spathulipetala* exhibits divergence in the form of petals. For some specimens, only the fourth and fifth petals become filiform (Fig. 5A), while for others, exclusively the fifth (Fig. 5B). Petal aestivation type is cochlear (Fig. 5B). For some individuals of *T. spathulipetala*, the fifth petal overlaps the fourth (Fig. 5A). In both species, petals present simple trichomes on both abaxial and adaxial sides (Fig. 5B).

In the androecium, the double whorl of stamens develops in a manner similar to that of *T. paratyensis* in a unidirectional pattern (Fig. 4D; Fig. 5C). The anthers of the antesepalous stamens begin their expansion before the closure of the carpel cleft, followed by the anthers of the antepetalous stamens (Fig. 5D). At the mature anthers, it is possible to detect an expansion of the dome-shaped connective, which is more apparent in *T. denudata* (Fig. 5E). The carpel begins its formation, together with the antesepalous stamens (Fig. 4D), and the carpel cleft is adaxial (Fig. 4E), as also observed for *T. paratyensis*. After its closure, the carpel starts to expand and curve towards the adaxial region of the flower bud (Figs. 5E and 5F). Simple trichomes are observed throughout the carpel (Fig. 4F; Figs. 5C–5F).

## Discussion

### Floral ontogeny

Although the floral morphology of the genus *Tachigali* has already been described in several taxonomic and morphological studies (van Der Werff, 2008; Silva, 2007; Silva & Lima, 2007; Silva et al., 2016; Casanova et al., 2020), only limited data are available about the early floral development of this group which presents small and large flowers with a wide variability of structures and symmetry (van Der Werff, 2008; Casanova et al., 2020). In the present study, the ontogenetic analysis of representative species of the genus provides insight into the early development of *Tachigali* flowers. Moreover, the comprehension of the floral varieties, symmetry, and homologies within the group.

Flower buds of *Tachigali* revealed an acropetal and helical development. This aspect is frequently observed for Caesalpinoideae (Tucker, 2003) and for some Papilionoideae (Leite et al., 2015), mainly in racemose, paniculate and umbel inflorescences (Tucker, 2003). This pattern has also been reported for the genus by Casanova et al. (2020). The flower buds develop at the axils of the bracts, which provide protection during the early stages. However, the bracts are known to fall into senescence before anthesis. For these species, the formation of bracteoles could not be observed, albeit some authors, such as Reis et al. (2015) and Silva et al. (2016), report the presence of such structures on the flowers of the genus, also indicating the presence of bracteoles in *T. spathulipetala*. The presence of minimal bracts, or their complete absence, is also observed for the genus *Tachigali* of the clade Tachigali, as well as other related genera, such as *Dimorphandra* Schott, *Diptychandra* Tul., and the mimosoid clade (Barros et al., 2017).

For all studied species, the sepals revealed a helical pattern of development with the first sepal in the abaxial position. This frequent characteristic of Caesalpinoideae is also observed in some Papilionoideae (Tucker, 1984, 2003; Bruneau et al., 2014). However, the position of the second, fourth, and fifth sepals of *T. paratyensis* stands in contrast to that of *T. denudata* and *T. spathuliepetala* (Table 1). This pattern has been described as “modified helical” and has also been reported for *Dimorphandra mollis* Benth. (Caesalpinoideae) with some modifications such that the first sepals also emerge in the abaxial region, but the other three develop in different positions (Barros et al., 2017). Moreover, for all species, the sepals present a quincuncial aestivation type, according to the classification of Endress (1994). This imbrication type, as well as the sepal development pattern, provides a zygomorphic character to the flower buds of the studied species.

In all species, petal formation is initiated only after the establishment of the sepals, as already reported for several leguminous species (Bruneau et al., 2014; Barros et al., 2017; Kochanovski et al., 2018). Furthermore, the petal whorl alternates with the sepal whorl during development. Nevertheless, unlike the other Caesalpinoideae in which the petals develop unidirectionally, with the first petal in adaxial position (Tucker, 2003; Bruneau et al., 2014), the petals of the species analyzed present a helical pattern of development with the first petal in abaxial position and the fifth in adaxial position for all species. The position of the other three petaloid primordia varies for *T. paratyensis* relative to *T. denudata* and *T. spathulipetala* (Table 1). Each of the species presents distinct petal morphology in the late stages of floral development. However, in the early stages, the petals morphology does not vary. In the early stages of flower bud development, the three species petaloid primordia varies in its order of initiation. The petal morphology has already been described in previous studies, as a relevant character for interspecific distinction (Silva, 2007; Silva & Lima, 2007; van Der Werff, 2008; Silva et al., 2016; Casanova et al., 2020). Additionally, T. *paratyensis* and *T. spathulipetala* present an imbricated corolla with cochlear aestivation type, according to Endress (1994). Both aestivation type and developmental pattern of the petals provide a zygomorphic character to the flower buds of *T. paratyensis* and *T. spathulipetala*, as well as described for the sepals. It was difficult to classify the aestivation type of *T. denutada* petals because they do not expand laterally, and their edges do not touch or overlap. However, the petals apex touches each other, also their trichomes, giving those petals a slightly cochlear aestivation type. Nevertheless, these petal aestivation type *T. denutada* is subtle compared to the other species studied, so the zygomorphy of the floral bud is mainly evidenced by the pattern of quincuncial aestivation of the sepals.

According to Ronse De Craene (2008) sepals and petals aestivation are not definitively correlated. It is known that imbricate petals, initiated in a spiral, thick and fleshy, have the same aestivation as the sepals. However, this is less evident when petals appear almost simultaneously or later in their development, as is the case of the *Tachigali* species analyzed here. The aestivation patterns can be variable even within a single family, as in Acanthaceae (Scotland, Endress & Lawrence, 1994), or even between species of the same genus, as observed in *Croton* (Thaowetsuwan et al., 2020). According to Thaowetsuwan et al. (2020), different patterns are often observed in imbrication type between calyx and corolla, which can follow different patterns, as in the case of *Tachigali*, since the pattern of aestivation of the sepals is different from the petals.

In the androecium, both whorls of stamens present unidirectional development, consisting of a frequently observed pattern among Caesalpinoideae (Bruneau et al., 2014). On the developed anthers, the presence of an extension of the dome-shaped connective can be seen. Both this characteristic and the presence of a double whorl of stamens have recently been described by Casanova et al. (2020) to the same species and highlighted as important aspects for genus diagnosis. Additionally, among analyzed species, the androecium presents an actinomorphic aspect during the early stages of development.

The carpel initiates its development in parallel with the antesepalous stamens, as also reported for *Dimorphandra mollis* (Caesalpinoideae) (Barros et al., 2017). The premature development of this structure is considered to be widespread among leguminous species (Prenner & Cardoso, 2017). The carpel cleft is positioned adaxially, as well as the carpel extension and the style curvature, an equally frequent characteristic of leguminous species, except for the subfamily Detarioideae, in which the style has an abaxial curvature (Prenner & Cardoso, 2017).

In addition, the all analyzed species might be distinguished by the timing of formation of trichomes on floral whorls. The presence of simple and glandular trichomes has already been described in several studies based on their relevance to the description of species in this genus (Silva, 2007; van Der Werff, 2008; Silva et al., 2016; Casanova et al., 2020). In the present study, differential timing of trichome establishment could be observed, varying among the analyzed species. For *T. paratyensis*, the trichomes of the sepals begin their formation before the complete settlement of this whorl, whereas the trichomes of the petals, the androecium and the gynoecium are formed later. Alternatively, for *T. denudata* and *T. spathulipetala*, the trichomes of the petals and carpel are the first to be formed. Only later are trichomes observed in the abaxial side of some petals, as already described by Casanova et al. (2020).

Regarding the floral morphology of the related genera in Tachigali clade, *Arapatiella* and *Jacqueshuberia* species have morphologically similar flowers within *Tachigali*. *Arapatiella* have flowers with tubular hypanthium, oval petals, unguiculate and stipulated ovary (Cowan, 1973; Silva, 1976; Cowan, 1981); while *Jacquehusberia* presents flowers with cupular hypanthium, ovate petals, and sessile ovary (Silva & Graham, 1980). Both genera have bigger flowers then observed in *Tachigali* (Silva et al., 2016).

### Flower symmetry

According to our results, the developmental patterns of sepals and petals interfere with the types of imbrications observed for the whorls. Those types, quincuncial and cochlear, respectively, according to the definitions given by Endress (1994), guarantee zygomorphy of the flower buds in early stages of development for all analyzed species. Casanova et al. (2020) describes the symmetry of the flowers of *Tachigali* based on the asymmetry observed in the hypanthium and the shape of the petals and stamens. *Tachigali paratyensis* is classified as strongly zygomorphic owing to its tubular and asymmetric hypanthium and its heteromorphic stamens (Watson & Dallwitz, 1983; Casanova et al., 2020), while *T. spathulipetala* is described as slightly zygomorphic by its asymmetric hypanthium and heteromorphic petals. *Tachigali denudata* is described as zygomorphic exclusively for its flower buds, also because of its asymmetric hypanthium, but actinomorphic after anthesis owing to isomorphic petals and stamens (Casanova et al., 2020). Our discussion of flower symmetry of the genera is corroborated by data related to the zygomorphic and premature development of the sepals and petals.

As for the other genus in *Tachigali* clade, only flowers of *Jachusberia* are slightly zygomorphic (Watson & Dallwitz, 1983). There is no flower symmetry classification for *Arapatiella*, which needs mores morphological and developmental studies.

## Conclusions

This study is an unprecedented record of ontogeny in *Tachigali*. We found important characteristics in floral development of the species, such as sepals and petals with helical development, varying the position of the primordia in the bud, according to the different species; stamens with unilateral development and carpel with adaxial curvature. However, for *Arapatiella* and *Jaqueshuberia* it requires studies of this type to better understand the floral evolution of this diverse group. Here we also corroborate the discussion of the floral symmetry of the genus with data related to the zygomorphic and early development of the sepals and petals.

In this sense, with the present study, we corroborate the circumscription of the genus *Tachigali* based on shared ontogenetic characters. Future studies in conjunction with a more robust phylogeny of the genus allowed further analysis of the intraspecific relationships of the group.

## Supplemental Information

10.7717/peerj.13975/supp-1 Supplemental Information 1 Floral ontogeny of *Tachigali paratyensis*. (A- G) inflorescence axis at the top of the images. (A-D) bracts removed; (E- G) bracts and sepals removed. (A) floral primordium at the base of the bract; observe the formation of the first sepal (yellow) in the abaxial position. (B) two floral primordia with three sepals initiated (yellow 1-3); emergence of simple trichomes indicated by arrowhead. (C) three sepals in formation (yellow 1-3) and the primordia of the fourth (yellow 4) and fifth (yellow 5); simple trichomes indicated by arrowhead. (D) all sepals already set in the flower bud (yellow 1-5). (E) first sepal (yellow 1) removed, the other sepals (yellow 2-5) are arranged as quincuncial aestivation pattern. The dashline indicated the position of the five sepal under the sepals 2 and 3. (F) five petals in formation (green 1-5), as well as the first stamen in abaxial position (purple 1) and the second stamen primordia (asterisk) carpel development initiation (orange). (G) establishment of five petals (green 1-5) and antesepalous stamens (purple 1-3); carpel in development (orange). Bar: A-B, D-E = 20 µm; C, F = 50 µm.

10.7717/peerj.13975/supp-2 Supplemental Information 2 Floral ontogeny of *Tachigali paratyensis*. (A-C) inflorescence axis at the top of the images. (A-B) bracts and sepals removed. (C-F) bracts, sepals and petals removed; (G) bracts, sepals, petals, and stamens removed. (A) establishment of petals (green 1-5) and antesepalous whorl of stamens (purple 1-3); carpel in development (orange) with emergence of the carpel cleft (arrowhead) and the first antepetalous stamen primordium (blue). (B) petals expanding (green 1-5) and carpel elongating (orange) with the closure of the carpel cleft (arrowhead), and antepetalous stamens primordia (blue 4). (C) development of antesepalous stamens (purple 1-3) and antepetalous stamens primordia (blue 4-6); carpel elongating (orange). (D) stamen development (purple 1-3; blue 4-6); carpel curvature (orange) towards the adaxial portion. (E) established double whorl of stamens (purple 1-3; blue 4-6); style bent towards the adaxial portion (orange). (F) side view of floral bud; arrowhead indicating the double whorl of stamens (purple and blue). (G) side view of the flower bud showing only the gynoecium (orange). Bar: A-D = 100 µm; E = 500 µm; F-G = 200 µm.

10.7717/peerj.13975/supp-3 Supplemental Information 3 Floral ontogeny of *Tachigali denudata* (A-F). (A-F) inflorescence axis at the top of images. (A-C) bracts removed. (D-F) bracts and sepals removed. (A) floral primordium at the base of the bract; observe the primordia of the first (S1) and second sepal (S2). (B) emergence of the third (S3), fourth (S4) and fifth (S5) sepals. (C). five sepals; development of trichomes in the abaxial surface of the sepals (arrowhead). (D) primordia of the five petals (P1-P5); primordia of the antesepalous stamens (A1-A3); carpel development initiation (C). (E) filiform petals established; carpel in development (C) with emergence of the carpel cleft (arrowhead). (F) filiform petals established (P1-P5); carpel elongating (C) with closure of the carpel cleft. Bar: a-b, d = 20 µm; c = 100 µm.

10.7717/peerj.13975/supp-4 Supplemental Information 4 Floral ontogeny of *Tachigali spathulipetala* (A–D) and *Tachigali denudata* (E,F). (A-D) inflorescence axis at the top of images. (A-B) bracts and sepals removed. (C-E) bracts, sepals and petals removed. (F) bracts, sepals, petals, and stamens removed. (A-B) filiform and spatulate petals (green 1-5); carpel in development (orange), and an antepetalous stamen primordium (blue 6). (C) antepetalous stamens established (purple 1-3); antepetalous stamen primordia (blue 4-6); carpel in development (orange) with emergence of carpel cleft. (D) anthers of the an te sepalous stamens expanding (purple 1-3); carpel (orange) elongating; and antepetalous stamen primordia (blue 4-6). (E) anthers already formed on both androecium whorls (purple 1-3, blue 4-6); connective extension (arrowhead style bent towards the adaxial portion. (F) side view of the flower bud, only the gynoecium (orange); arrowhead pointing to stigma. Bar: A-D = 100 µm; E-F = 200 µm.

## Additional Information and Declarations

### Data Availability

The following information was supplied regarding data availability: The raw data is available in the Supplemental Files.
