# Supplementary figures and images for: Floral ontogeny of Tachigali (Caesalpinioideae, Fabaceae) species
Source: PeerJ. 2022 Sep 8;10:e13975. doi: 10.7717/peerj.13975 (PMC9464433; doi:10.7717/peerj.13975)

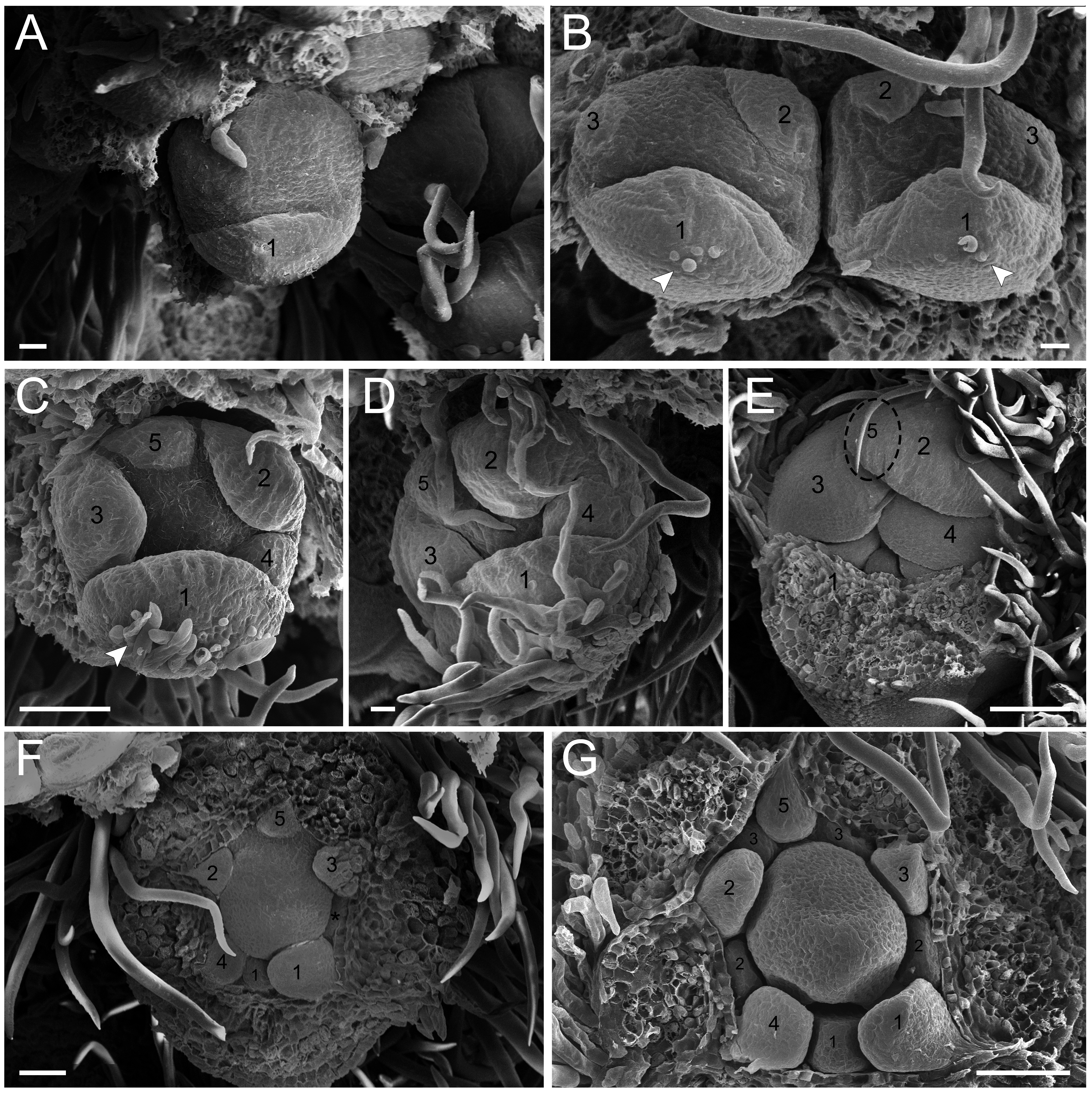

Supplement: Supplemental Information 1 — (A- G) inflorescence axis at the top of the images. (A-D) bracts removed; (E- G) bracts and sepals removed. (A) floral primordium at the base of the bract; observe the formation of the first sepal (yellow) in the abaxial position. (B) two floral primordia with three sepals initiated (yellow 1-3); emergence of simple trichomes indicated by arrowhead. (C) three sepals in formation (yellow 1-3) and the primordia of the fourth (yellow 4) and fifth (yellow 5); simple trichomes indicated by arrowhead. (D) all sepals already set in the flower bud (yellow 1-5). (E) first sepal (yellow 1) removed, the other sepals (yellow 2-5) are arranged as quincuncial aestivation pattern. The dashline indicated the position of the five sepal under the sepals 2 and 3. (F) five petals in formation (green 1-5), as well as the first stamen in abaxial position (purple 1) and the second stamen primordia (asterisk) carpel development initiation (orange). (G) establishment of five petals (green 1-5) and antesepalous stamens (purple 1-3); carpel in development (orange). Bar: A-B, D-E = 20 µm; C, F = 50 µm. [file peerj-10-13975-s001.jpg]

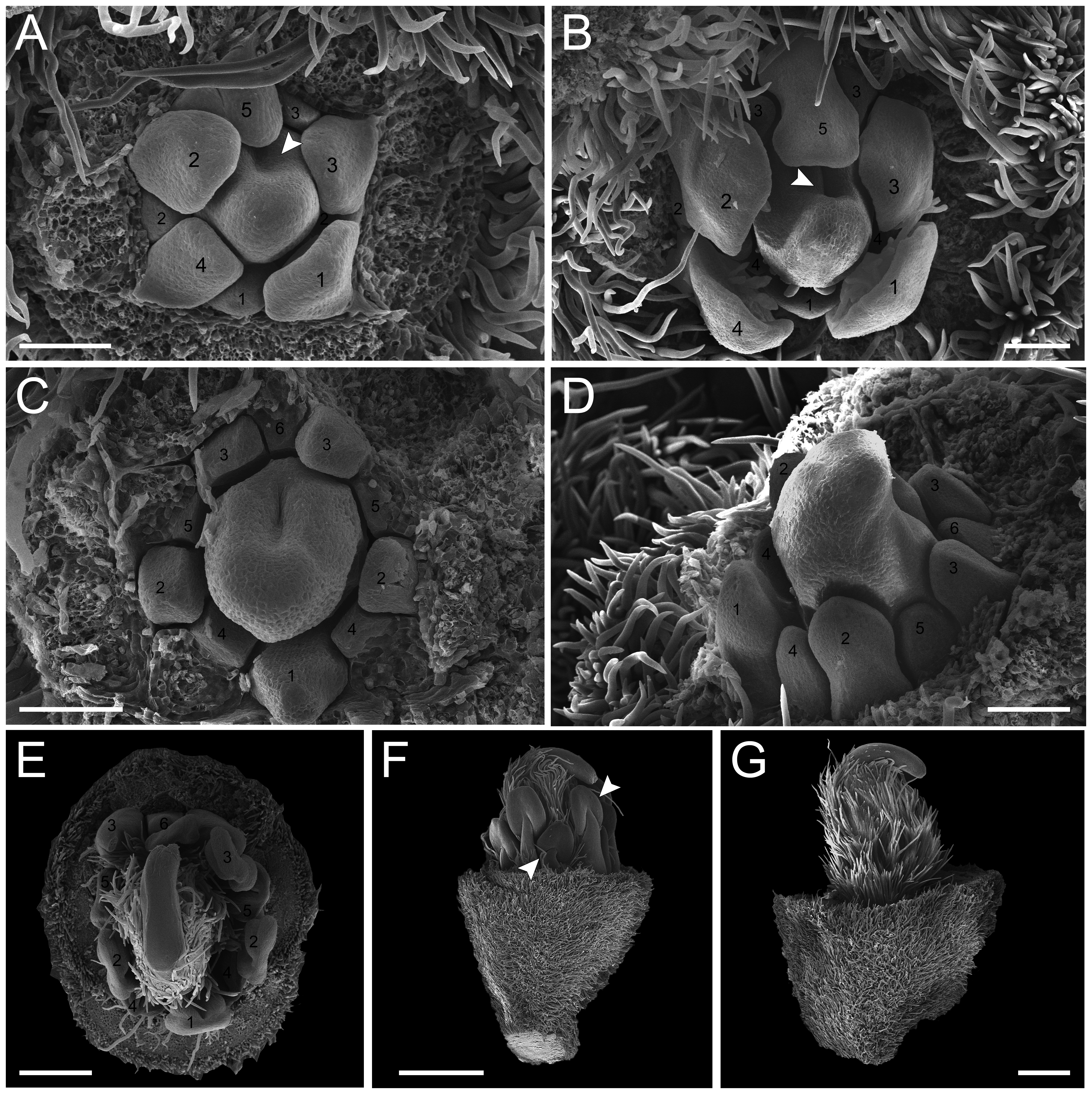

Supplement: Supplemental Information 2 — (A-C) inflorescence axis at the top of the images. (A-B) bracts and sepals removed. (C-F) bracts, sepals and petals removed; (G) bracts, sepals, petals, and stamens removed. (A) establishment of petals (green 1-5) and antesepalous whorl of stamens (purple 1-3); carpel in development (orange) with emergence of the carpel cleft (arrowhead) and the first antepetalous stamen primordium (blue). (B) petals expanding (green 1-5) and carpel elongating (orange) with the closure of the carpel cleft (arrowhead), and antepetalous stamens primordia (blue 4). (C) development of antesepalous stamens (purple 1-3) and antepetalous stamens primordia (blue 4-6); carpel elongating (orange). (D) stamen development (purple 1-3; blue 4-6); carpel curvature (orange) towards the adaxial portion. (E) established double whorl of stamens (purple 1-3; blue 4-6); style bent towards the adaxial portion (orange). (F) side view of floral bud; arrowhead indicating the double whorl of stamens (purple and blue). (G) side view of the flower bud showing only the gynoecium (orange). Bar: A-D = 100 µm; E = 500 µm; F-G = 200 µm. [file peerj-10-13975-s002.jpg]

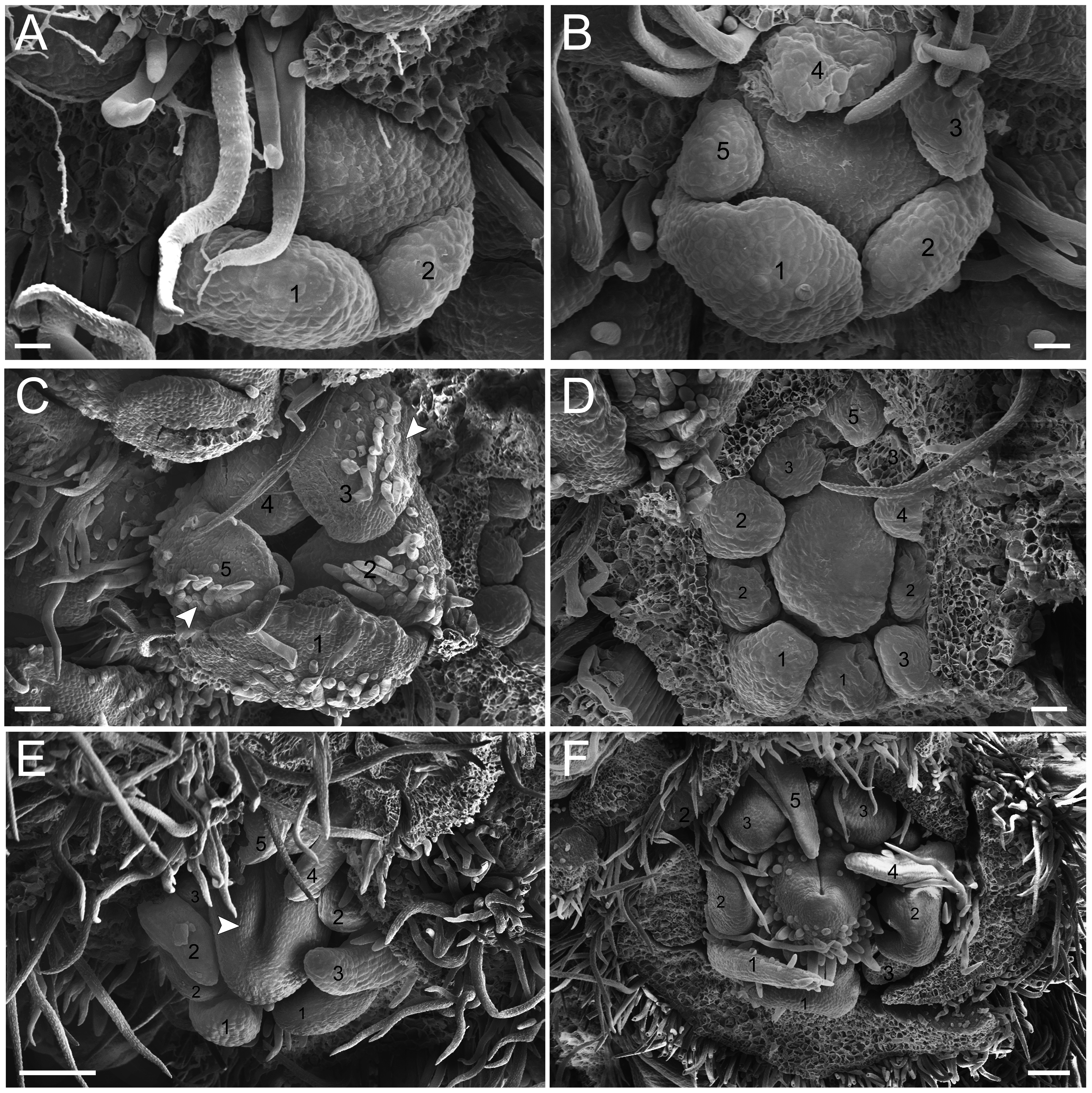

Supplement: Supplemental Information 3 — (A-F) inflorescence axis at the top of images. (A-C) bracts removed. (D-F) bracts and sepals removed. (A) floral primordium at the base of the bract; observe the primordia of the first (S1) and second sepal (S2). (B) emergence of the third (S3), fourth (S4) and fifth (S5) sepals. (C). five sepals; development of trichomes in the abaxial surface of the sepals (arrowhead). (D) primordia of the five petals (P1-P5); primordia of the antesepalous stamens (A1-A3); carpel development initiation (C). (E) filiform petals established; carpel in development (C) with emergence of the carpel cleft (arrowhead). (F) filiform petals established (P1-P5); carpel elongating (C) with closure of the carpel cleft. Bar: a-b, d = 20 µm; c = 100 µm. [file peerj-10-13975-s003.jpg]

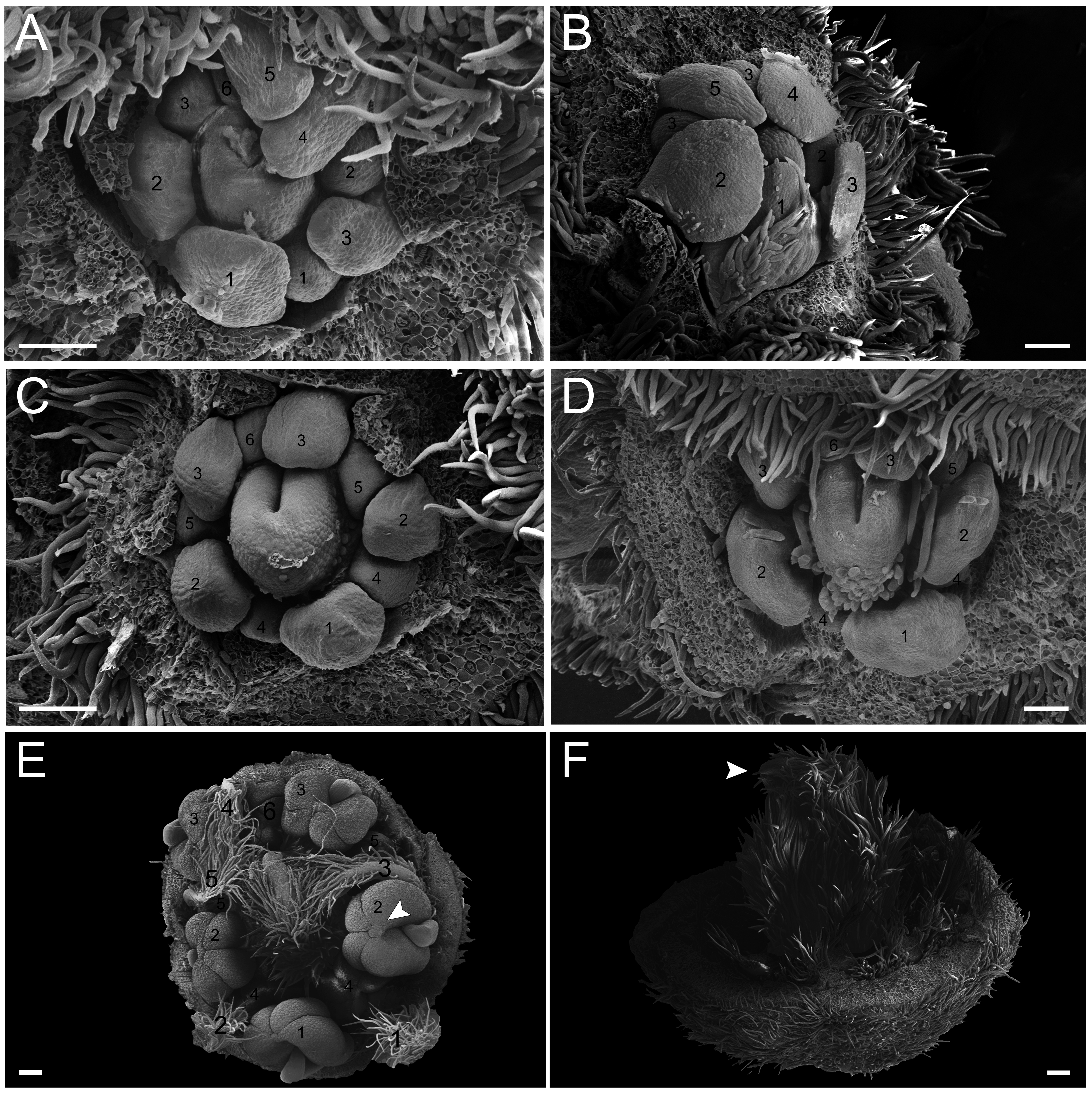

Supplement: Supplemental Information 4 — (A-D) inflorescence axis at the top of images. (A-B) bracts and sepals removed. (C-E) bracts, sepals and petals removed. (F) bracts, sepals, petals, and stamens removed. (A-B) filiform and spatulate petals (green 1-5); carpel in development (orange), and an antepetalous stamen primordium (blue 6). (C) antepetalous stamens established (purple 1-3); antepetalous stamen primordia (blue 4-6); carpel in development (orange) with emergence of carpel cleft. (D) anthers of the an te sepalous stamens expanding (purple 1-3); carpel (orange) elongating; and antepetalous stamen primordia (blue 4-6). (E) anthers already formed on both androecium whorls (purple 1-3, blue 4-6); connective extension (arrowhead style bent towards the adaxial portion. (F) side view of the flower bud, only the gynoecium (orange); arrowhead pointing to stigma. Bar: A-D = 100 µm; E-F = 200 µm. [file peerj-10-13975-s004.jpg]
